# Variability of *in vivo* Sarcomere Length Measures in the Upper Limb Obtained With Second Harmonic Generation Microendoscopy

**DOI:** 10.3389/fphys.2021.817334

**Published:** 2022-02-08

**Authors:** Amy N. Adkins, Ryan M. Fong, Julius P. A. Dewald, Wendy M. Murray

**Affiliations:** ^1^Department of Biomedical Engineering, Northwestern University, Evanston, IL, United States; ^2^Shirley Ryan AbilityLab, Chicago, IL, United States; ^3^Edward Hines, Jr. VA Hospital, Hines, IL, United States; ^4^Department of Physical Therapy and Human Movement Sciences, Northwestern University Feinberg School of Medicine, Chicago, IL, United States; ^5^Department of Physical Medicine and Rehabilitation, Northwestern University Feinberg School of Medicine, Chicago, IL, United States

**Keywords:** sarcomere length, second harmonic generation imaging, skeletal muscle, muscle architecture, sarcomere variability

## Abstract

The lengths of a muscle’s sarcomeres are a primary determinant of its ability to contract and produce force. In addition, sarcomere length is a critical parameter that is required to make meaningful comparisons of both the force-generating and excursion capacities of different muscles. Until recently, *in vivo* sarcomere length data have been limited to invasive or intraoperative measurement techniques. With the advent of second harmonic generation microendoscopy, minimally invasive measures of sarcomere length can be made for the first time. This imaging technique expands our ability to study muscle adaptation due to changes in stimulus, use, or disease. However, due to past inability to measure sarcomeres outside of surgery or biopsy, little is known about the natural, anatomical variability in sarcomere length in living human subjects. To develop robust experimental protocols that ensure data provide accurate representations of a muscle’s sarcomere lengths, we sought to quantify experimental uncertainty associated with *in vivo* measures of sarcomere lengths. Specifically, we assessed the variability in sarcomere length measured (1) within a single image, along a muscle fiber, (2) across images captured within a single trial, across trials, and across days, as well as (3) across locations in the muscle using second harmonic generation in two upper limb muscles with different muscle architectures, functions, and sizes. Across all of our measures of variability we estimate that the magnitude of the uncertainty for *in vivo* sarcomere length is on the order of ∼0.25 μm. In the two upper limb muscles studied we found larger variability in sarcomere lengths within a single insertion than across locations. We also developed custom code to make measures of sarcomere length variability across a single fiber and determined that this codes’ accuracy is an order of magnitude smaller than our measurement uncertainty due to sarcomere variability. Together, our findings provide guidance for the development of robust experimental design and analysis of *in vivo* sarcomere lengths in the upper limb.

## Introduction

Whole muscle is made up of hundreds of thousands of sarcomeres arranged in series and parallel. The length of a muscle’s sarcomeres is a primary determinant of a muscle’s ability to contract and produce force (Gordon et al., 1966). In addition, when quantifying muscle architecture, sarcomere length is a critical parameter that is required to make meaningful comparisons of both the force-generating and excursion capacities of different muscles. With the advent of second harmonic generation (SHG) microendoscopy for measuring sarcomere length minimally invasively (Llewellyn et al., 2008), there are exciting and novel opportunities to measure sarcomere length *in vivo*.

Anatomical studies in cadavers, which involve dissection and measurement across multiple scales (whole muscles, fascicles, and sarcomeres), demonstrate that a major distinction among different skeletal muscles in the human body is the number of sarcomeres in series and parallel (Brand et al., 1981; Lieber et al., 1990; Lieber and Fridén, 2000). Serial sarcomere number (SSN) is a particularly important architectural parameter, as it describes the range of lengths over which a muscle can actively generate force and, given optimal sarcomere length, a measure of the fascicle length at which the muscle will produce its maximum isometric force. The number of parallel sarcomeres is proportional to the maximum isometric force a muscle can produce. In anatomical studies, SSN is commonly characterized by a muscle’s optimal fascicle length (OFL), which is calculated as the ratio of fascicle and sarcomere lengths measured from a muscle’s dissected fascicles, multiplied by optimal sarcomere length. Similarly, the number of sarcomeres in parallel is characterized by a muscle’s physiological cross-sectional area (PCSA), which is calculated from the ratio of the muscle’s volume and OFL, with a correction for the pennation angle of the fibers to estimate how much of the force-generating capacity is transmitted by the tendon. Measures of *in vivo* fascicle length and muscle volume have been made using safe and effective imaging techniques for decades. The novel capacity to make minimally invasive measures of sarcomere length *in vivo* provides new opportunities to study functionally meaningful muscle parameters (OFL and PCSA) and how they vary in living subjects across muscles, individuals, and due to alterations in muscle stimulus or use.

Due to the novelty of methods for sarcomere length measurement that do not involve surgery or biopsy, little is known about the natural, anatomical variability in sarcomere length in living human subjects. Such an understanding is critical for the development of robust experimental protocols that ensure data provide accurate representations of a muscle’s sarcomere lengths. Animal muscle studies (e.g., Moo et al., 2016, 2017) and a single *in vivo* study on the tibialis anterior (Lichtwark et al., 2018) suggest that sarcomere length can vary considerably throughout a muscle. Importantly, the SHG microendoscopy method provides only a small field-of-view (82 μm × 82 μm, ∼20–35 sarcomeres in series) (Llewellyn et al., 2008; Sanchez et al., 2015). In addition, current protocols for processing SHG data utilize a method which provides a single measure—the mean sarcomere length—per image, precluding information about within fiber variability. To enable effective studies of how muscle sarcomere length may differ or change (e.g., across limbs, after an exercise intervention, following an injury, etc.), more information describing the natural anatomical variability, both across different locations in a single muscle and along a single muscle fiber, is needed.

In this study we aim to quantify experimental uncertainty associated with *in vivo* measures of sarcomere lengths made using second harmonic generation microendoscopy in two muscles of the upper limb (biceps brachii and flexor carpi ulnaris). Given the precision of the SHG imaging and measurement procedures established in the literature is small (∼30 nm) (Sanchez et al., 2015), we expect the primary sources of measurement uncertainty to include both natural anatomical variability of the lengths of the sarcomeres within these muscles and the random error associated with test-retest reliability using this method. In this study, we assessed the variability in sarcomere length measured (1) within a single image, along a muscle fiber, (2) across images captured within a single trial, across trials, and across days, as well as (3) across locations in the muscle. Quantifying the degree of experimental uncertainty for sarcomere length measures made using second harmonic generation microendoscopy will aid interpretation of these novel data, guide the design of future work aimed at detecting sarcomere length differences among different populations, and enable error propagation when sarcomere length measures are incorporated with other anatomical measures to calculate functionally meaningful muscle architectural parameter values.

## Materials and Methods

### Data Collection

The 14 participants enrolled in this study provided informed consent; Northwestern University’s Institutional Review Board approved this study’s procedures. Imaging for both the biceps brachii and flexor carpi ulnaris (FCU) was completed under passive conditions, with participants seated. For biceps imaging, the participants arm was placed with the shoulder abducted 85°, elbow extended 25°, and wrist at neutral (0°), as verified by handheld goniometric measures. For the FCU, the limb was positioned with the elbow at 70° of flexion and the wrist at neutral (0°). Soft velcro straps were used to secure the arm after the joint angles were verified.

To image sarcomeres *in vivo*, a microendoscopic probe consisting of two 1.8 cm long, 20-gauge needles with beveled tips was inserted into the muscle of interest with its optical lenses aligned parallel to the fascicle direction. Insertion location of the probe was guided through palpation techniques and ultrasound imaging in the imaging limb posture. Ultrasound images were viewed in both the short and long axis planes of the muscle to identify key anatomical landmarks and to ensure both insertion in the correct muscle of interest and avoidance of major blood vessels, nerves, and bone. Once established, the appropriate location for probe insertion was marked on the skin.

As described previously (Adkins et al., 2021), a spring loaded injector was used to insert the microendoscopic probe into the muscle. Once inserted, images are acquired 0.3 cm superficial to the tip of the probe. Differences in muscle size and body composition among different participants means the exact depth that images are collected within the muscle can vary between subjects. For both the biceps brachii and FCU, we aimed to obtain images away from the inner tendon. For the biceps brachii, the distal nature of the inner tendon and the dimensions of the muscle and subcutaneous fat observed among our participants meant we could accomplish this while the full length of the probe needles (1.8 cm) were inserted in the muscle. Due to the longer central tendon, the smaller muscle belly size of the FCU, and its close proximity to the ulnar nerve, we needed to limit the insertion depth of the microendoscopic probe. For FCU, we modified imaging depth at the time of injection by adding custom spacers (thickness ranged from 0.2 to 0.7 cm) between the external surface of the skin and the base of the spring loaded injector.

Once the probe was placed as desired within the muscle belly, a second-harmonic generation microscope (Enspectra Health, Mountain View, CA, United States) was attached to it for imaging. In this implementation [probe design same as (Lichtwark et al., 2018; Adkins et al., 2021) as opposed to older design (Sanchez et al., 2015; Chen and Delp, 2016; Chen et al., 2016)], laser light travels down one of the probe’s needles, interacts with the muscle tissue, and is then received by the other needle. The resulting images capture the intrinsic striation pattern (A-bands) of sarcomeres and have a field of view of 82 × 82 microns. Data were collected at 1.9 Hz for approximately 2–5 min (∼250–600 images per acquisition). The system was calibrated by the manufacturer using a precision Ronchi Ruling target with 10-micron line spacing to calculate the conversion factor (0.16 microns per pixel) for quantification of striation spacing from the images.

### Variability Along Fiber

Current image processing methods for analysis of SHG images (mean sarcomere length code; MSLC) report a single mean sarcomere length value for an entire image (e.g., Sanchez et al., 2015; Adkins et al., 2021). Here, we developed and validated an image processing and analysis code (individual sarcomere length code: ISLC) which quantifies: the lengths of individual sarcomeres within an image, the mean length of these sarcomeres, and standard deviation in sarcomere length within the image. To verify the performance of the ISLC, realistic virtual phantoms were designed, tested for their similarity to real data, and then used to calculate and compare the accuracy and precision of the novel ISLC and the existing MSLC.

#### Individual Sarcomere Length Code

**FIGURE 1:**
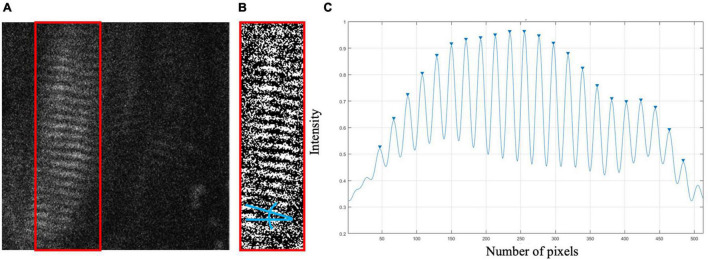
**(A)** Sample raw SHG image of *in vivo* biceps brachii sarcomere data (512 pixels × 512 pixels, 82 μm × 82 μm). Canny edge detection selected region enclosed by red box. **(B)** Binarized selected region with blue lines indicating the angle relative to horizontal. **(C)** Pixel vs. intensity distribution where A-bands are represented by peaks.

The image processing protocol developed in this study was designed to quantify the length of individual sarcomeres within an image, along the imaged fiber direction, using custom written MATLAB code. Specifically, the ISLC takes a Fast Fourier Transform (FFT) of each image and applies a threshold to reduce noise and clear the edges of the Fourier space. The inverse FFT (IFFT) is computed, and Canny edge detection is utilized to select the largest area in the image with sarcomeres (Figure 1A). Within the selected region, the fiber orientation is determined by binarizing the region and calculating the orientation relative to the horizontal axis of the box (Figure 1B). Sarcomere length is calculated along a single column of pixels as the pixel distance between pixel intensity peaks (A-bands), converted to microns (0.16 μm per pixel) and multiplied by the cosine of the detected angle to correct for angle of fiber orientation (Figure 1C). This analysis process is repeated for distinct columns of pixels at 2 μm intervals to enable the collection of sarcomere length data from separate myofibrils (Lieber, 2002; Moo et al., 2016).

#### Realistic Virtual Phantoms

**FIGURE 2:**
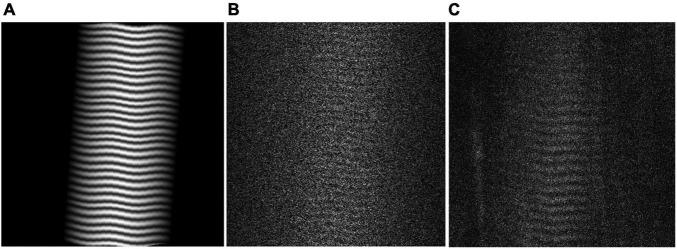
**(A)** Virtual Phantom created with real sarcomere lengths and variability, distortion, and rotation. **(B)** Realistic Virtual Phantom with added noise. **(C)** Representative raw data from *in vivo* biceps brachii.

Virtual phantoms were designed to closely mimic raw, *in vivo* data. A range of phantoms with sarcomere length averages and standard deviations representative of cadaveric data from the biceps brachii [data from Murray et al. (2000)] were generated using a series of normally distributed sinusoids (Figure 2A). To mimic observed raw SHG data, the image signal in the phantom was degraded by removing 70% of the pixels at random. A sinusoidal geometric transformation was applied to create fiber bending, and Gaussian white noise was added to capture similar signal quality (Figures 2B,C). To assess the phantoms’ similarity to real data, the signal-to-noise ratio (SNR) for the phantoms was measured and then adapted to mimic the range of SNR values found in raw SHG images from the biceps brachii.

#### Determining Accuracy and Comparing Individual Sarcomere Length Code and Mean Sarcomere Length Code

Using 300 virtual phantom images with known sarcomere lengths, and varying image quality, the code’s accuracy and precision were determined by comparing the ISLC output to the known phantom values. The accuracy and precision of the ISLC code developed in this study was defined from the differences between the “true” mean sarcomere lengths defined in the virtual phantoms and the mean sarcomere length values output from the ISLC. Accuracy was defined by the difference in means; precision was defined by the standard deviation of the differences. This analysis was repeated for the MSLC to enable direct comparison of the two methods. A Bland-Altman test was implemented to determine agreement between sarcomere lengths obtained using ISLC and sarcomere measurements obtained using MSCL.

### Variability Across Images, Trials, and Days

Sarcomere length variability across different images from a single imaging trial (defined as a single needle insertion and microscope attachment) was assessed using the standard deviation of the mean sarcomere length from all processed images in the trial. For this study, we reanalyzed images originally collected for a separate study (Adkins et al., 2021) that were acquired in the non-paretic biceps of 7 individuals with chronic hemiparetic stroke and in the biceps from both the dominant and non-dominant limbs of 4 individuals with no neuromuscular impairments (Table 1). Therefore, SHG images were collected from the long head of the biceps brachii in 15 limbs. On average, we collected 145 ± 108 images per trial. Mean sarcomere length per image was calculated using MSLC.

**TABLE 1:** Participant demographic and participation information.

	Arm	Gender	Age	Stroke	Muscle	Location	Across images	Across trials	Across days	Across locations
*1	L	F	65	X	BB	1	X			
*2	R	F	63	X	BB	1	X			
*3	R	M	48	X	BB	1	X			
*4	L	M	60	X	BB	1	X			
*5	L	M	72	X	BB	1	X			
*6	R	M	62	X	BB	1	X			
*7	L	M	44	X	BB	1	X	X		
	R							X		
8	R	F	65	X	BB	1			X	
	L								X	
*9	R	F	53		BB	1	X			
	L						X			
*10	R	F	64		BB	1	X			
	L						X			
*11	R	M	62		BB	1	X			
	L						X			
*12	R	M	67		BB	1	X			
	L						X	X		
13	R	M	29		BB	4		1X	4X	4X
	L							1X		4X
	R				FCU	2				2X
	L									2X
14	L	F	28		BB	4				4X
		6F/8M	56 (14)	Total Comparisons			15	5	6	16

*Age for all participants is reported as of the time at which the experimental data collection for that participant was completed.*

*The average and standard deviation of “Age” is given in the last row as “average (standard deviation).” For “Muscle”—BB stands for Biceps Brachii and FCU stands for Flexor Carpi Ulnaris.*

*Indicates original data was collected from Adkins et al. (2021) and reanalyzed in this study for the purpose of understanding sarcomere variability.

Images collected from 3 participants were analyzed to evaluate the variability in sarcomere lengths measured when two trials of data were collected in the same muscle (Table 1, different trials arise from a single needle insertion, but distinct microscope attachment). Across trials, there is a slight change in location or orientation of the probe within the muscle due to the removal and reattachment of the microscope on the probe. In some cases, we also apply slight pressure or other movements to the needle between trials. Because the needle is not removed and reinserted, we expect we are likely sampling from the same relatively small, localized area, but from different sarcomeres due to the probe movement. In total, we analyzed two trials of data collected from 5 distinct insertions, including insertions in both arms of two participants, and a single arm from a third participant. Variability between 2 trials of image data was quantified as the difference in the average sarcomere length per trial (i.e., the average value of the output of MSLC for all the images in that trial).

The variability in sarcomere lengths measured on different days (a minimum of 1 week apart) was assessed in the long head of the biceps brachii. Images collected from a single needle insertion in both limbs of 1 participant and from 4 insertion sites (at different locations in the muscle) from a single limb in a second participant were analyzed (Table 1). Because measurements were taken on different days, the location of the probe within the muscle across days is sensitive to replicability of arm position (which would effect the muscle-tendon length the images were collected in), identification of the location in the muscle where the needle should be inserted, and the process of probe insertion. In these evaluations, the investigator’s intention was to perform each of these tasks consistently across days. Variability in the sarcomere lengths measured on 2 different days was quantified as difference in the average sarcomere length captured on “Day 1” and “Day 2” (i.e., the average value of the output of MSLC for all the images in the single trial of data collected from a given needle insertion location each day). In four of the repeated measures across days, the insertion location was marked on the limb and the distance between insertion locations was measured and determined to be less than 1 cm apart (average ± standard deviation, 0.54 ± 0.17 cm).

### Variability in Different Locations in the Muscle

**FIGURE 3:**
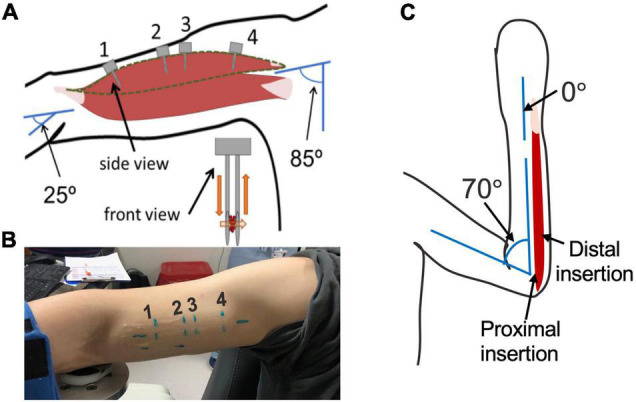
**(A)** schematic of arm position and location of probe insertion in the long head of the Biceps Brachii. Illustration of probe showing the direction of laser light and interaction with muscle tissue (orange). **(B)** Picture of location of four probe insertions in one of the study participants. **(C)** Schematic of the elbow and wrist position and locations of the probe insertion in the flexor carpi ulnaris.

**FIGURE 4:**
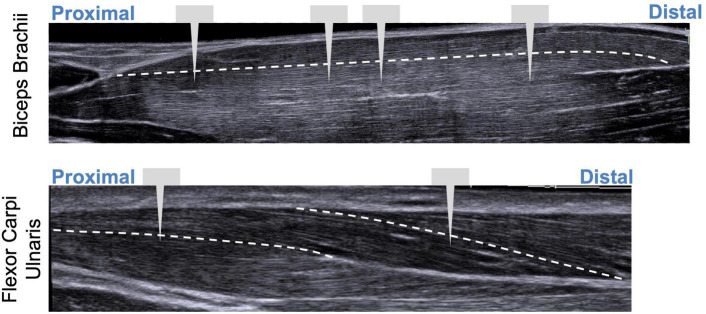
Illustration showing extended field-of-view ultrasound images with approximate locations of probe insertions along the muscle. White dashed line represents fascicle paths.

Sarcomere lengths in the biceps brachii were measured at distinct locations in the muscle in three arms of two individuals (1 female, 28 years; 1 male, 26 years) (Figure 3). Four insertions were made; insertions near the distal and proximal ends of the muscle belly (position 1 and 4), and two insertions in the center of the muscle belly approximately 1 cm apart (positions 2 and 3). In both arms of a single individual (male, 29 years old) sarcomere length measures were made at two locations along the flexor carpi ulnaris muscle; a proximal location where the fibers run longitudinally in the muscle, and a distal location where the fibers are pennated (Figure 3). Measurements of fascicle length of the long head of the biceps brachii and the flexor carpi ulnaris were obtained using extended field-of-view ultrasound (Acuson S2000 Ultrasound System, Siemens Medical Solutions United States, Inc., Mountain View, CA, United States) at the same joint posture sarcomere length was obtained (Figure 4). Ultrasound images were exported as uncompressed DICOM files and fascicles were measured using the segmented line tool in ImageJ (Wayne Rasband, National Institutes of Health, Bethesda, MD, United States).

## Results

### Variability Along Fiber

**FIGURE 5:**
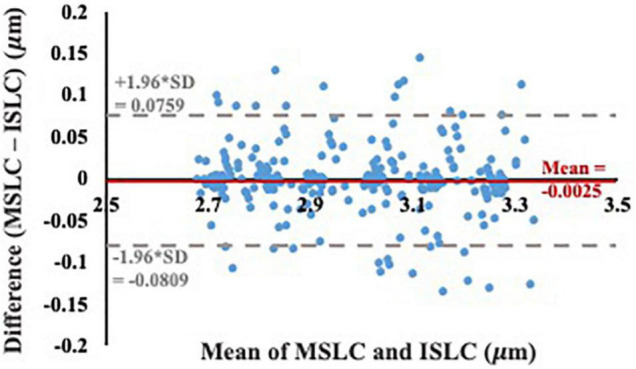
Bland-Altman test of agreement between our individual sarcomere length code (ISLC) and the image processing method currently utilized in the literature, MSLC. The x-axis is the average of the MSLC and ISLC sarcomere length measurements and the y-axis is the difference between the sarcomere lengths calculated from the two methods. The solid red line indicates the bias (–0.0025 μm) and the dashed gray lines represent the lower (–0.0809 μm) and upper (0.0759 μm) limits-of-agreement (mean difference ± 1.96*standard deviation of the difference).

The ISLC code developed in this study was accurate to 0.02 μm and precise to 0.02 μm. While the analysis from the virtual phantoms indicates the MSLC code was slightly less accurate than the ISLC (accuracy 0.03 μm, precision 0.02 μm), the Bland-Altman analysis (Figure 5) demonstrates that mean sarcomere lengths that result from the two analyses are comparable. There is a minimal bias between the two methods (−0.0025 μm; 95% limits-of-agreement = −0.0809–0.0759 μm), there is no observable systematic variance, and the measurements lay within the limits-of-agreement.

### Variability Across Images, Trials, and Days

The mean sarcomere length observed in a single image varied across the multiple images that are captured in a single imaging trial (i.e., single needle insertion and microscope attachment), although this variability is relatively small. Across 15 trials, the standard deviation of the mean sarcomere length per image within a trial was 0.20 ± 0.07 μm. The magnitude of the difference in mean sarcomere length measured in different trials (average ± standard deviation: trials: 0.18 ± 0.10 μm) was comparable to the variability across images and the difference in mean sarcomere length measured in the same muscle on different days (0.15 ± 0.13 μm). When standard deviations of sarcomere lengths within a single image were analyzed (from a single participant across all locations), variability within that image were on the same order (0.13 ± 0.02 μm) as those we report across images. Given this, and the fact the phantom study identified the ISLC and MSLC were of comparable accuracy, the MSLC was used for all *in vivo* sarcomere length data.

### Variability Across Muscle

**FIGURE 6:**
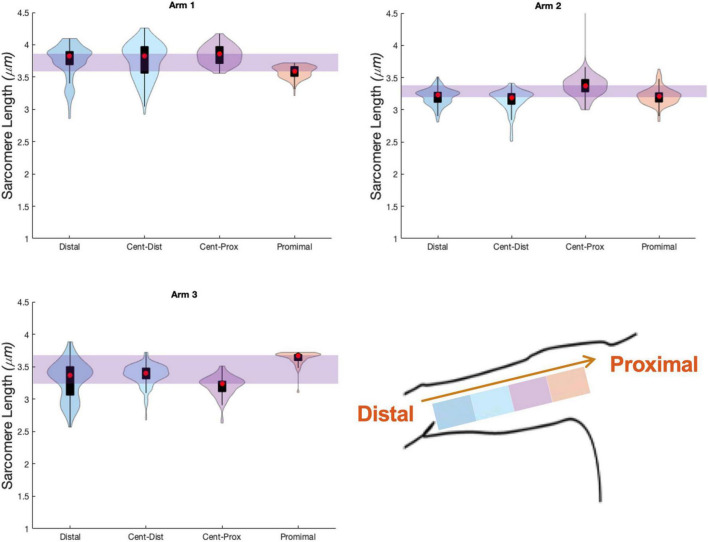
Violin plots for 4 locations, and three arms showing median of data (red circle), interquartile range (black box plot), and probability density of data at each location (shaded violin shape). Arm 2 (right, dominant) and Arm 3 (left, non-dominant) are from a single individual.

**FIGURE 7:**
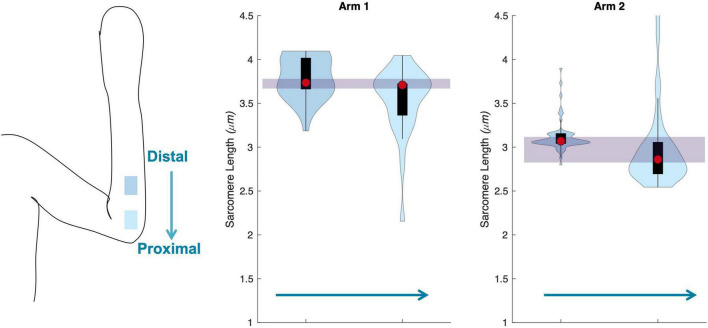
Schematic of the two insertion locations (left) in the flexor carpi ulnaris., and three arms showing median of data (red circle), interquartile range (black box plot), and probability density of data at each location (shaded violin shape).

The uncertainty in sarcomere length that was introduced by sampling from various, distinct muscle locations was not larger than the variability in mean sarcomere length we observed across images within a single trial (i.e., needle insertion). For the biceps brachii, the difference in median sarcomere length across four locations was 0.15 ± 0.12 μm. For the flexor carpi ulnaris, the difference in median sarcomere length across two locations was 0.12 ± 0.13 μm. Standard deviations of images collected in a single trial and location averaged 0.18 ± 0.07 μm for the biceps and 0.34 ± 0.17 μm for the FCU. The largest difference in median sarcomere length between any two locations in a single muscle occurred in the biceps brachii of arm 3 between the proximal and center-proximal location (difference = 0.43 μm). Notably, for both muscles and for all arms evaluated, the maximum difference in median sarcomere length observed at different locations (c.f. Figures 6, 7 purple shaded region) was less than the range of sarcomere lengths observed in a single stick (colored probability distribution of each violin plot for each arm).

### Summary/Functional Implications

**FIGURE 8:**
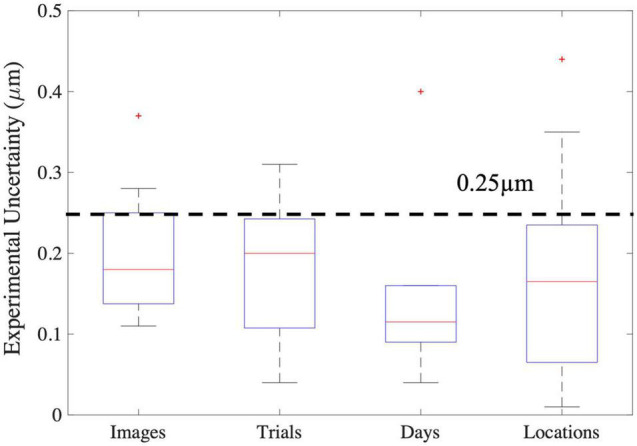
Boxplots of the various sources of experimental uncertainty investigated in this study. The red line represents the median, the blue box extends from the 25th to the 75th percentile of the data, and the dashed error bars, referred to as whiskers, represent the range of all data excluding outliers (red “+”). For images the y-axis represents the standard deviation in mean sarcomere length across images. For all other x-axis elements, the y-axis represents the absolute difference in mean sarcomere length between trials, days, and locations.

Based on the sources of variability in sarcomere length measures in the upper limb muscles investigated in this study, we estimate the uncertainty (a lumped quantity that includes both the natural anatomical variability of sarcomere lengths within the muscle and experimental/random error) in sarcomere length associated with collecting SHG microendoscope images in a single imaging session from a single needle insertion within the muscle to be on the order of 0.25 μm (Figure 8). Because the variability observed across trials, probe insertions, and different days was not systematically larger than the variability observed in a single trial, we expect 0.25 μm, which was ∼7–8% of the average sarcomere lengths in the biceps brachii and FCU, provides a reasonable measure of natural anatomical variability of sarcomere lengths for these two muscles. Notably, this level of variability is an order of magnitude greater than the improvement in measurement accuracy obtained if the variability in sarcomere length within an image is considered along a muscle fiber (difference in accuracy between ISLC and MCLC ∼0.01μ*m*). Given it is used in the calculation of functionally relevant architectural parameters, the uncertainty in sarcomere length measures we observed would yield uncertainty in calculations of optimal fascicle length and physiological cross-sectional area of ∼7–8%, independent of any experimental uncertainty in measurements of fascicle length or muscle volume.

## Discussion

We investigated both anatomical and experimental sources of variability in sarcomere length measures obtained *in vivo*, in the upper limb, using second harmonic generation microendoscopy. Based on assessments completed in two muscles in the upper limb that have different functions, size, and architectures, we estimate the magnitude of the uncertainty in such measures to be on the order of ∼0.25 μm, which we conclude is dominated by the anatomical variability of sarcomere lengths in these muscles, under these conditions, rather than from random error associated with our methods. Notably, we found that, in both a fusiform muscle (biceps brachii) and pennate muscle (flexor carpi ulnaris) of the upper limb, variability in sarcomere length within a single insertion is larger than the variability across locations in these muscles. We developed custom image processing code which takes measures of individual sarcomeres in an image, allowing for the quantification of sarcomere length variability along a muscle fiber and across myofibrils in a localized area (82 μm by 82 μm). We found that the variability in mean sarcomere length measures made using this ISLC was comparable to current processing methods (MSLC) and an order of magnitude smaller (∼0.02 μm) than the uncertainty in sarcomere length associated with all other sources investigated in this study (i.e., across images, trials, days, locations). Together, our findings provide guidance for the development of robust experimental design and analysis of *in vivo* sarcomere lengths in the upper limb.

The uncertainty in sarcomere length observed in this study (<8%), and its propagation through calculation of the most functionally meaningful muscle parameters (optimal fascicle length and PCSA), is smaller than differences reported in *in vivo* sarcomere length and fascicle length of various studies in the literature. This finding suggests that using SHG to detect *in vivo* sarcomere length adaptation is practical. For example, intraoperative measures of sarcomeres have been shown to be much longer in children with cerebral palsy (an injury of the brain during development) than sarcomere length estimates for Mathewson et al. (2015) (∼88%) or intraoperative measures (Lieber and Fridén, 2019) (∼46%) in typically developing children. In addition, following 3 weeks of Nordic hamstring training, a 17% increase in sarcomere length was found in the biceps femoris long head (Pincheira et al., 2021). Due to the relative ease of obtaining fascicle length measures as compared to sarcomere length measures *in vivo*, many studies have reported adaptation of fascicle length to altered use or stimulus. Previous studies in elbow flexors of individuals with chronic hemiparetic stroke report substantial decreases in fascicle length in extended joint postures [∼19% decrease in biceps brachii at 25° elbow flexion (Nelson et al., 2018), 15% decrease in brachialis at 10° elbow flexion (Li et al., 2007)]. Fascicle length in gastrocnemius medialis in individuals who regularly wear high heels were found to be 11% shorter than non-high heel wearers (Csapo et al., 2010). If these changes in fascicle length were due solely to changes in sarcomere length (i.e., a decrease in sarcomere length of 10–20%), our study suggests SHG microendoscopy would be able to reliably detect such changes. Notably, in a single individual who underwent a leg lengthening surgery (4 cm distraction) to correct a limb discrepancy, sarcomere length decreased by ∼15% while fascicle length increased by over 100% (Boakes et al., 2007). Together, these changes resulted in an overall increase in serial sarcomere number of 134.6% (Boakes et al., 2007), emphasizing the importance of measurement of both fascicle and sarcomere length to fully understand muscle function and plasticity.

Our data suggest the approach of measuring sarcomere lengths from a single insertion site in the muscle belly is appropriate for an understanding of average sarcomere length and variability in a fusiform (biceps brachii) and pennate (flexor carpi ulnaris) muscles of the upper limb. This finding will guide the development of future studies determining interlimb or interindividual differences in sarcomere length in the biceps brachii or flexor carpi ulnaris. For example, an *a priori* power analysis to determine the difference in sarcomere length between two dependent means (e.g., left and right arm) using SHG microendoscopy (standard deviation of 0.25 micrometers) would demonstrate that a power over 0.8 would be achieved with 10 participants with a detectable difference of 0.25 micrometers (effect size of 1) between limbs.

The mean sarcomere lengths observed in this study (3.04–3.97 μm) are substantially longer than optimal length (2.6–2.8 μm)(Gordon et al., 1966). These lengths are likely best explained by the joint postures in which data were collected, and that data collection occurs while the muscle is passive. In other studies in living human subjects, similar sarcomere lengths were observed under similar conditions. Most notably, in two different intraoperative studies, the native (prior to surgical tendon transfer) *in vivo* sarcomere lengths of the brachioradialis with the elbow fully extended were 3.90 ± 0.27 μm [*n* = 8; (Lieber et al., 2005)] and 3.7 ± 0.3 μm [*n* = 12; (Murray et al., 2006)]. Similarly, sarcomere lengths comparable to those we report here were reported for other muscles while the joint they crossed was positioned such that muscle-tendon lengths were relatively long. These *in vivo* studies include both intraoperative laser diffraction [e.g., extensor carpi radialis brevis lengths ranged from 3.1–3.9 μm in different participants (Lieber et al., 1994)] and *in vivo* microendoscopic imaging [e.g., soleus ∼3.0–4.2 μm (Chen and Delp, 2016), tibialis anterior 2.5–3.9 μm, (Lichtwark et al., 2018)]. Notably, when joint posture was repositioned in these same studies, SL decreased, and the length ranges included optimal length in these new joint postures [2.4–3.0 μm (Lieber et al., 1994), ∼2.5–3.5 μm (Chen and Delp, 2016), 2.3–3.8 μm, (Lichtwark et al., 2018)]. Sarcomere lengths substantially longer than optimal length have also been observed in the long head of the biceps brachii in cadaveric specimens (Murray et al., 2000). Finally, it is important to interpret these data with the understanding that data collection occurred while the muscles were passive. Due to the series elastic properties of muscle and tendon, sarcomere lengths will shorten to some degree during active force generation (Zajac, 1989).

In this study, we did not observe systematic differences in sarcomere lengths from the images collected from probe insertions at different locations along the length of the muscle belly. In isolated muscle fiber experiments, markedly shorter sarcomeres have been observed within single fibers at the at the extreme ends of the fibers, close to the fiber-tendon junction (Huxley and Peachey, 1961). Using these methods, the lengths of sarcomeres can be quantified at multiple locations in a single fiber, with the precise location of the measurement expressed as a displacement relative to a reference point on the fiber. Given the precision of these methods, data can also be collected very close to the fiber-tendon junction. Data in Huxley and Peachey (1961) suggest the shorter sarcomere lengths were only observed at a distance from the end of the fiber that was approximately ∼5% of total fiber length. While we sampled from very different locations in the muscle belly (cf., Figure 3), given the rigidity and size of the microscope, the geometry of the muscle belly near the muscle-tendon junction, and the lack of control we have in placing the probe precisely in the muscle belly using this imaging technique, we neither intended nor do we expect that we sampled from such an extreme end of the muscle belly. Other studies in which the sampling methodology is more similar to our methods than single fiber experiments (e.g., Moo et al., 2016) also present data without the systematic effects observed close to the muscle-tendon junction in single fiber data. The data we present here describe the level of uncertainty associated with methodologies where random samples of sarcomere lengths are measured from different locations within a muscle’s belly and will provide important guidance for interpretation of sarcomere length data measured *in vivo* in human participants.

This study provides insight which will be invaluable for the design, implementation, and analysis of future *in vivo* architectural studies aiming to explore muscle plasticity. However, there are several limitations in this work which should be considered. In particular, the relatively small cohort of individuals we evaluated limited our ability to make statistical conclusions. In addition, we only explore sarcomere length variability in a small number of muscles and at a single joint posture and contraction state per muscle. Future studies in these directions would be of interest. Notably, most previous *in vivo* muscle architectural studies demonstrate differences in fascicle length without normalizing to sarcomere length or make estimates of PCSA by using this non-normalized fascicle length. With the addition of sarcomere length differences across populations, following interventions, or impairment of the two most functionally relevant parameters of muscle (serial sarcomere length and PCSA) can be explicitly calculated. This work provides confidence in the ability to use SHG microendoscopy to make measures of *in vivo* sarcomere length to detect difference in functionally relevant muscle parameters on the order of 8% or greater.

## Data Availability Statement

The data supporting the conclusions of this article will be made available by the authors in Arch, Northwestern University Research and Data Repository (doi: 10.21985/n2-8mwv-mz5).

## Ethics Statement

The studies involving human participants were reviewed and approved by Northwestern University Institutional Review Board. The patients/participants provided their written informed consent to participate in this study.

## Author Contributions

AA, RF, JD, and WM designed the research, performed the research, and wrote the manuscript. AA, RF, and WM analyzed the data. All authors contributed to the article and approved the submitted version.

## Author Disclaimer

Any opinions, findings, and conclusions or recommendations expressed in this material are those of the authors and do not necessarily reflect the views of the NSF, NIH, or DeLuca Foundation.

## Conflict of Interest

The authors declare that the research was conducted in the absence of any commercial or financial relationships that could be construed as a potential conflict of interest.

## Publisher’s Note

All claims expressed in this article are solely those of the authors and do not necessarily represent those of their affiliated organizations, or those of the publisher, the editors and the reviewers. Any product that may be evaluated in this article, or claim that may be made by its manufacturer, is not guaranteed or endorsed by the publisher.
